# A Cognition-Based Method to Ease the Computational Load for an Extended Kalman Filter

**DOI:** 10.3390/s141223067

**Published:** 2014-12-03

**Authors:** Yanpeng Li, Xiang Li, Bin Deng, Hongqiang Wang, Yuliang Qin

**Affiliations:** School of Electrical Science and Engineering, National University of Defense Technology, 137 Yanwachi Street, Changsha 410073, China; E-Mails: lixiang01@vip.sina.com (X.L.); dengbin@nudt.edu.cn (B.D.); oliverwhq@tom.com (H.W.); yuliang.qin@gmail.com (Y.Q.)

**Keywords:** computational load, extended Kalman filter, target localizing, target tracking

## Abstract

The extended Kalman filter (EKF) is the nonlinear model of a Kalman filter (KF). It is a useful parameter estimation method when the observation model and/or the state transition model is not a linear function. However, the computational requirements in EKF are a difficulty for the system. With the help of cognition-based designation and the Taylor expansion method, a novel algorithm is proposed to ease the computational load for EKF in azimuth predicting and localizing under a nonlinear observation model. When there are nonlinear functions and inverse calculations for matrices, this method makes use of the major components (according to current performance and the performance requirements) in the Taylor expansion. As a result, the computational load is greatly lowered and the performance is ensured. Simulation results show that the proposed measure will deliver filtering output with a similar precision compared to the regular EKF. At the same time, the computational load is substantially lowered.

## 1. Introduction

In statistics and parameter estimation, the Kalman filter is a mathematical method named after Rudolf E. Kalman [1,2]. Its purpose is to use observations (measurements), which are observed over time and that contain noise (random processes) and other inaccuracies, to arrive at values that tend to be closer to the true values of the observations and their associated calculated values [1,3].

The Kalman filter is a very useful facility in parameter estimation, target tracking and data fusion [1,4]. One of its conveniences is that, when the observation noise (or measure noise) is Gaussian, the predicting covariance matrix, the filtering gain and the filtering covariance matrix can be calculated offline [1,5]. An example is as follows. In radar target tracking, if the target's state equation and the sensor's measure equation are either linear in the same coordinate and the measure noise is Gaussian with zero mean, then the Kalman filter algorithm is fairly brief, and the request for minimum variance is easy to meet [6,7].

However, the Kalman filter needs a linear observation model, which may be impractical in some circumstances [7,8]. To address the problem when observation models become nonlinear, researchers developed sub-optimal extensions of a Kalman filter. In these methods, the extended Kalman filter (EKF) is the nonlinear version of a Kalman filter [9,10]. It linearizes the estimation of the current mean and covariance [11]. In the scenario of well-defined transition models, the EKF has been considered to be a practical means of nonlinear state estimation [7,12].

The literature on the Kalman filter and EKF is extensive. In distributed sensor and/or collaborative sensor networks, the Kalman filter and EKF are frequently employed in parameter estimation and target tracking [13,14]. For instance, to smooth a tractor global positioning system (GPS) that is sharpened when the tractor moves over rough terrain, an improved Kalman filter is proposed [13]. The novel filter can satisfactorily preprocess the low-cost GPS receiver data [13]. In distributed state estimation for wireless sensor network, when the system process model and observation model are nonlinear, it is proven that the distributed extended Kalman filter can provide an identical state estimation of the system state [15,16]. For estimating the three-dimensional orientation of a rigid body, a quaternion-based EKF is developed [17,18]. Another example shows that EKF is effective at target tracking in the presence of glint noise [19]. The extended Kalman filter can also be employed to an online training forward neural network applied to time series predicting [20]. It is proven that this nonlinear filtering method has some advantages, such as handling additive noises and following the movement of a system when the underlying model is evolving through time [20].

Based on the aforementioned materials, it can be seen that EKF has been widely used. Unfortunately, the computational load is a bottleneck in the implementation of an extended Kalman filter [21]. Due to the limited resources and heavy clutter, this problem may be complicated for an airborne radar system or a space-borne radar system. Since most of the EKF has a nonlinear observation model (as is also true in this work), linearization of the nonlinear observation model is continuously investigated [11]. The work of linearization is greatly affected by a varying scenario, and this raises the computational load when it generates a statistically optimal estimation of the real system state [4]. Therefore, algorithms to lower the computational cost have been addressed [4].

It is clear that the extra computational load brought to EKF compared to a Kalman filter is linearizing the nonlinear observation model in the above-mentioned situation. Therefore, most of these methods introduce the Taylor expansion technology [4,22,23]. The nonlinear observation model is expressed by the Taylor expansion. The frequently-used approach is a one-order Taylor expansion [22,24]. In simple scenarios, it is beneficial for lowering the computational requirements (our previous work showed that the Taylor expansion technology is also helpful for lowering the computational load for a Kalman filter [5]). However, due to the fixed designation, the actual precision will be scenario dependent. For some complicated scenarios (or in complicated processing steps), the output performance may be non-ideal. For smooth points, some of the calculations may be unnecessary. The filter is thus not flexible.

On the other side, cognitive radar and knowledge-aided signal processing are investigated [25,26]. A fully-cognitive radar (active radar) can adapt both its receiver and transmitter in response to statistical variations in the environment in real time [25]. Therefore, it can meet specific remote-sensing objectives in an efficient, reliable and robust manner [25]. For an active radar, there are many available works on target tracking based on cognitive processing [27–29]. However, for a passive radar, the existing work focusing on target tracking based on cognitive processing is limited.

In view of the above-mentioned facts, this work investigates a novel fast EKF method when there is a nonlinear observation model (particularly for a passive radar) [5]. The contributions are as follows. With the help of cognitive processing, a new fast EKF method is proposed. The items calculated in the Taylor expansion of the filtering gain and the localizing steps depend on the filtering performance at the last step. As a result, this designation saves time and computational resources. At the same time, it offers an adjustable performance. The designation of a fast EKF algorithm is thus perfected.

This paper is arranged as follows. Firstly, the problem and its background have been analyzed. The key ideas of this work are explained. These are the main contents in Section 1. The rest of the paper is organized as follows:

- The variables and their definitions are collected in Section 2.
- Section 3 details the processing techniques of an extended Kalman filter.
- The majority of our work presents the novel designation for an EKF. The cognition idea and the algorithm are further detailed in Section 4.
- Simulation results and the analysis of them are shown in Section 5.
- In Section 6, a simple summary is provided.

## 2. Nomenclature

Variables related to the target's state and observation:

**x**(*k*) = [*x*(*k*) *ẋ*(*k*) *y*(*k*) *ẏ*(*k*)]*^T^* is the target's state. It is a vector (2 × 1), which consists of the position and the velocity in the XoY plane.
**z**(*k*) = [*θ*(*k*) *θ̇*(*k*)]*^T^* + **w**(*k*) is the observation. It is a vector (2 × 1), which consists of the azimuth and the rate of change in the azimuth of the target. The observation noise is included.
**w**(*k*) is the observation noise introduced by the sensor and the propagation effects. It is a vector (2 × 1) in this work. After linearizing the observation model, the observation noises are **w**_0_(*k*) and **w**_1_(*k*).
Δ*t* is the data acquisition period.

Variables related to predicting and filtering:

**x̂**(*k* + 1∣*k*) is the predicting output (by the filter) of the target's state. It is a vector (4 × 1) in this work.
**x̂**(*k*∣*k*) is the filtering output. It is a vector (4 × 1) in this work.
***ν***(*k* + 1) is the innovation. It is a vector (2 × 1) in this work.
**R**(*k*) is the covariance matrix of the observation noise. It is a matrix with 2 × 2 entries in this work.
**P**(*k* + 1∣*k*) is the covariance matrix of the predicting noise. It is a matrix with 4 × 4 entries in this work.
$\hat{\theta }(k)={\left[\hat{\theta }(k)\;\;\hat{\dot{\theta }}(k)\right]}^{T}$ is the predicting output (by the filter) of the target's azimuth and the rate of change in the azimuth. It is a vector (2 × 1) in this work.
*ϕ*(*k* + 1, *k*) is the transition matrix from time *k* to time *k* + 1. It is a matrix with 4 × 4 entries in this work.
**C**(*k* + 1) is the observation matrix. It is a matrix with 2 × 4 entries in this work. Different algorithms may have different expressions for the observation matrix. They are named **C**_0_(*k* + 1) and **C**_1_(*k* + 1), respectively.
**K**(*k* + 1) is the filtering gain at time *k* + 1. It is a matrix with 4 × 2 entries in this work.

Variables related to performance evaluation:

For predicting the target's azimuth and the rate of change in the azimuth:

**D**_predict_(*k*) is the difference between the predicting output and the observation.
*P*_predict_ (*k*) is the predicting performance.
***η***_predict_ = [*η*_11_ *η*_12_]*^T^* is the performance bound for predicting.

For estimating the target's state:

**D**_localize_(*k*) is the difference between the filtering output and the localizing result via the observation.
*P*_localize_(*k*) is the localizing performance.
***η***_localize_ = [*η*_21_ *η*_22_ *η*_23_ *η*_24_]*^T^* is the performance bound for localizing.

Temporal variables:

There are some temporal variables (*ψ*_1_(*k*), *ψ*_2_(*k*), *ς*, *ξ*, *etc.*) defined to facilitate the mathematical description. They are not collected here.

## 3. The Extended Kalman Filter

As previously mentioned, the EKF is an approximate solution that allows us to extend the Kalman filtering idea to nonlinear observation models [30,31]. The EKF is presented with a classical target tracking application in this work. In signal processing, target localizing is performed, and the target's azimuth (orientation) is predicted.

### 3.1. The State Vector and the Observation Model

In particular, the state vector and the observation model take the form:

(1) $$\begin{array}{ll} \text{state vector} & \text{x}\left(k\right)\in {\text{R}}^{4}\\ \text{observation model} & \text{z}\left(k\right)=f\left(\text{x}\left(k\right)\right)+\text{w}\left(k\right) \end{array}$$

where **w**(*k*) is the observation noise introduced by the sensor and the propagation effects. The target here is a point-object (point target) moving in a two-dimensional plane. The target's state vector, **x**(*k*), consists of the position and the velocity in the plane:

(2) $$\text{x}\left(k\right)={\left[\begin{array}{cccc} x\left(k\right) & \dot{x}\left(k\right) & y\left(k\right) & \dot{y}\left(k\right) \end{array}\right]}^{T}.$$

However, only the azimuth and the rate of change in the azimuth of the target are observed by the employed passive radar. Thus,

(3) $$\begin{array}{ll} \text{z}\left(k\right) & ={\left[z_{1}\left(k\right)\;z_{2}\left(k\right)\right]}^{T}\\ & =f\left(\text{x}\left(k\right)\right)+\text{w}\left(k\right)\\ & ={\left[f_{1}\left(\text{x}\left(k\right)\right)\;f_{2}\left(\text{x}\left(k\right)\right)\right]}^{T}+\text{w}\left(k\right)\\ & ={\left[\theta \left(k\right)\;\dot{\theta }\left(k\right)\right]}^{T}+\text{w}\left(k\right). \end{array}$$

### 3.2. The Observation Geometry

**Figure 1.**
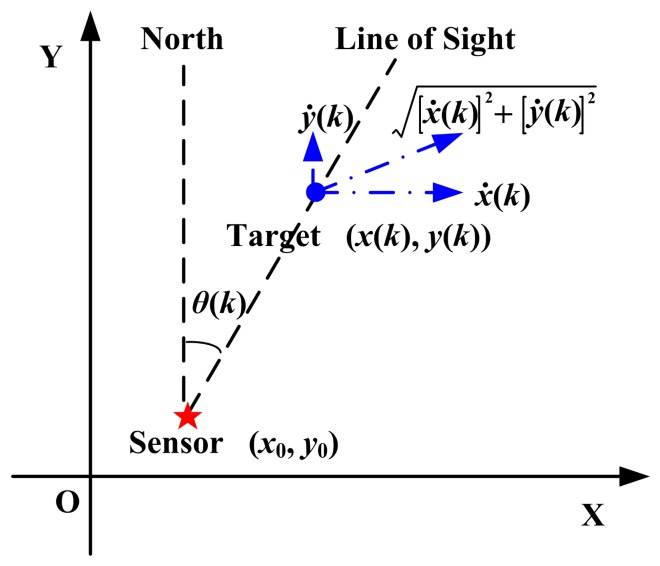
The radar-target geometry.

The radar-target geometry is shown in Figure 1. There is no barrier between the radar and the target.

According to Figure 1,

(4) $$\theta \left(k\right)=\arctan\left(\frac{x\left(k\right)-x_{0}}{y\left(k\right)-y_{0}}\right)$$

and:

(5) $$\dot{\theta }\left(k\right)=\frac{\dot{x}\left(k\right)\left(y\left(k\right)-y_{0}\right)-\dot{y}\left(k\right)\left(x\left(k\right)-x_{0}\right)}{{\left(x\left(k\right)-x_{0}\right)}^{2}+{\left(y\left(k\right)-y_{0}\right)}^{2}}.$$

With no loss of generality, the radar's coordinates are set to (0, 0) in the XoY plane. Therefore,

(6) $$\theta \left(k\right)=\text{arctan}\left(\frac{x\left(k\right)}{y\left(k\right)}\right)$$

and:

(7) $$\dot{\theta }\left(k\right)=\frac{\dot{x}\left(k\right)y\left(k\right)-\dot{y}\left(k\right)x\left(k\right)}{{\left(x\left(k\right)\right)}^{2}+{\left(y\left(k\right)\right)}^{2}}.$$

### 3.3. The Predicting and Filtering Steps

The one-step predicting algorithm and the filtering algorithm are as follows.

One-step predicting:

(8) $$\hat{\text{x}}\left(k+1|k\right)=\phi \left(k+1,k\right)\hat{\text{x}}\left(k|k\right)$$

where **x̂**(*k* + 1∣*k*) and **x̂**(*k*∣*k*) are the predicting output and the filtering output, respectively. *ϕ*(*k* + 1, *k*) is the transition matrix from time (step) *k* to time *k* + 1.

Innovation:

(9) $$\nu \left(k+1\right)=\text{z}\left(k+1\right)-\text{C}\left(k+1\right)\hat{\text{x}}\left(k+1|k\right)$$

where ***ν***(*k* + 1) is the innovation. **C**(*k* + 1) is the observation matrix.

Filtering:

(10) $$\hat{\text{x}}\left(k+1|k+1\right)=\hat{\text{x}}\left(k+1|k\right)+\text{K}\left(k+1\right)\nu \left(k+1\right)$$

where **K**(*k* + 1) is the filtering gain at time *k* + 1.

Determination of parameters and variables:

The transition matrix is a known matrix:

(11) $$\phi \left(k+1,k\right)=\left[\begin{array}{cccc} 1 & \Delta t & 0 & 0\\ 0 & 1 & 0 & 0\\ 0 & 0 & 1 & \Delta t\\ 0 & 0 & 0 & 1 \end{array}\right]$$

where Δ*t* is the data acquisition period.

The observation matrix is the linearization solution of the nonlinear observation model, and:

(12) z(k)=C(k)x(k)+w(k).

There are three kinds of EKF algorithms in this paper: the normal EKF, a fast EKF and a cognition-based fast EKF. The observation matrix for these algorithms is **C**_0_(*k*), **C**_1_(*k*) and **C**_2_(*k*), respectively. According to the performance evaluation result, **C**_2_(*k*) equals **C**_0_(*k*) or **C**_1_(*k*). In the following texts, the normal EKF (referred to as “EKF-normal”) is the conventional algorithm developed at the very beginning of EKF. The existing fast EKF is implemented according to [32]. The cognition-based fast EKF is the newly proposed method in this work. In the following text, the existing fast EKF may be referred to as “EKF-fast1,” while the proposed fast EKF may be called “EKF-fast2.”

The covariance matrix of the observation noise is:

(13) $$\mathrm{cov}\left[\text{w}\left(k+1\right)\right]=E\left[\text{w}\left(k+1\right){\text{w}}^{T}\left(j+1\right)\right]=\text{R}\left(k+1\right)\delta_{kj}.$$

The covariance matrix of the predicting noise is defined by:

(14) $$\text{P}\left(k+1|k\right)=E\left\{\left[\text{x}\left(k+1\right)-\hat{\text{x}}\left(k+1|k\right)\right]\;{\left[\text{x}\left(k+1\right)-\hat{\text{x}}\left(k+1|k\right)\right]}^{T}\right\}.$$

Based on the aforementioned results, the gain matrix is given by:

(15) $$\text{K}\left(k+1\right)=\text{P}\left(k+1|k\right){\text{C}}^{T}\left(k+1\right){\left[\text{C}\left(k+1\right)\text{P}\left(k+1|k\right){\text{C}}^{T}\left(k+1\right)+\text{R}\left(k+1\right)\right]}^{-1}.$$

Equations (8)–(15) are the basic predicting and filtering steps of an EKF. These equations will be applied in different implements of an EKF, only substituting the corresponding variables into them.

### 3.4. Linearization of the Nonlinear Observation Model

#### 3.4.1. Taylor Expansion of the Nonlinear Observation Model

In order to linearize the observation model, the Taylor expansion method is introduced. Expanding the observation equation in Equation (3) into a Taylor around **x̂**(*k* + 1∣*k*) gives:

(16) $$\begin{array}{l} \text{z}\left(k+1\right)\\ =f\left(\text{x}\left(k+1\right)\right)+\text{w}\left(k+1\right)\\ =f\left(\hat{\text{x}}\left(k+1|k\right)\right)+\sum_{n=1}^{\infty }\left[{\frac{d^{n}f\left(\text{x}\left(k\right)\right)}{d\text{x}{\left(k\right)}^{n}}|}_{\text{x}\left(k\right)=\hat{\text{x}}\left(k+1|k\right)}{\left(\text{x}\left(k+1\right)-\hat{\text{x}}\left(k+1|k\right)\right)}^{n}\right]+\text{w}\left(k+1\right) \end{array}$$

It should be noted that the agreement is applied in Equation (16) as follows:

(17) $${\frac{d^{n}f\left(\text{x}\left(k\right)\right)}{d\text{x}{\left(k\right)}^{n}}|}_{\text{x}\left(k\right)=\hat{\text{x}}\left(k+1|k\right)}={\frac{d^{n}f\left(\text{x}\left(k+1\right)\right)}{d\text{x}{\left(k+1\right)}^{n}}|}_{\text{x}\left(k+1\right)=\hat{\text{x}}\left(k+1|k\right)},n=1,2,3,\ldots .$$

Equation (16) will be further utilized in linearizing the observation model. In this work, the derivatives considered are the first order derivative and the second order derivative:

(18) $$\frac{d^{n}f\left(\text{x}\left(k\right)\right)}{d\text{x}{\left(k\right)}^{n}}=\left[\begin{array}{cccc} \frac{\partial^{n}\theta \left(k\right)}{\partial x{\left(k\right)}^{n}} & \frac{\partial^{n}\theta \left(k\right)}{\partial \dot{x}{\left(k\right)}^{n}} & \frac{\partial^{n}\theta \left(k\right)}{\partial y{\left(k\right)}^{n}} & \frac{\partial^{n}\theta \left(k\right)}{\partial y{\left(k\right)}^{n}}\\ \frac{\partial^{n}\dot{\theta }\left(k\right)}{\partial x{\left(k\right)}^{n}} & \frac{\partial^{n}\dot{\theta }\left(k\right)}{\partial \dot{x}{\left(k\right)}^{n}} & \frac{\partial^{n}\dot{\theta }\left(k\right)}{\partial y{\left(k\right)}^{n}} & \frac{\partial^{n}\dot{\theta }\left(k\right)}{\partial \dot{y}{\left(k\right)}^{n}} \end{array}\right],n=1,2.$$

To ensure a more concise mathematical description, two temporal variables are assigned to the derivatives. This gives:

(19) $$\psi_{1}\left(k\right)={\frac{df\left(\text{x}\left(k\right)\right)}{d\text{x}\left(k\right)}|}_{\text{x}\left(k\right)=\hat{\text{x}}\left(k+1|k\right)}$$

and:

(20) $$\psi_{2}\left(k\right)={\frac{d^{2}f\left(\text{x}\left(k\right)\right)}{d\text{x}{\left(k\right)}^{2}}|}_{\text{x}\left(k\right)=\hat{\text{x}}\left(k+1|k\right)}$$

#### 3.4.2. The Observation Model in the Normal EKF

In Equation (16), ignoring higher powers (than second order) of **x**(*k* + 1) − **x̂**(*k* + 1∣*k*) results in:

(21) $$\begin{array}{l} \text{z}\left(k+1\right)\\ =f\left(\hat{\text{x}}\left(k+1|k\right)\right)+\psi_{1}\left(k\right)\left(\text{x}\left(k+1\right)-\hat{\text{x}}\left(k+1|k\right)\right)\\ +\psi_{2}\left(k\right){\left(\text{x}\left(k+1\right)-\hat{\text{x}}\left(k+1|k\right)\right)}^{2}+\text{w}\left(k+1\right) \end{array}$$

wherein (**x**(*k* + 1) − **x̂**(*k* + 1∣*k*))^2^ is a scalar multiplication. Then:

(22) $$\begin{array}{l} {\left(\text{x}\left(k+1\right)-\hat{\text{x}}\left(k+1|k\right)\right)}^{2}\\ =\left[\begin{array}{c} x\left(k+1\right)-\hat{x}{\left(k+1|k\right)}^{2}\\ {\left(\dot{x}\left(k+1\right)-\hat{\dot{x}}\left(k+1|k\right)\right)}^{2}\\ {\left(y\left(k+1\right)-\hat{y}\left(k+1|k\right)\right)}^{2}\\ {\left(\dot{y}\left(k+1\right)-\hat{\dot{y}}\left(k+1|k\right)\right)}^{2} \end{array}\right]\\ =\left[\begin{array}{c} x^{2}\left(k+1\right)-2\hat{x}\left(k+1|k\right)x\left(k+1\right)+{\hat{x}}^{2}\left(k+1|k\right)\\ {\dot{x}}^{2}\left(k+1\right)-2\hat{\dot{x}}\left(k+1|k\right)\dot{x}\left(k+1\right)+{\hat{\dot{x}}}^{2}\left(k+1|k\right)\\ y^{2}\left(k+1\right)-2\hat{y}\left(k+1|k\right)y\left(k+1\right)+{\hat{y}}^{2}\left(k+1|k\right)\\ {\dot{y}}^{2}\left(k+1\right)-2\hat{\dot{y}}\left(k+1|k\right)\dot{y}\left(k+1\right)+{\hat{\dot{y}}}^{2}\left(k+1|k\right) \end{array}\right]\\ =\left[\begin{array}{c} x^{2}\left(k+1\right)-2\hat{x}\left(k+1|k\right)x\left(k+1\right)\\ {\dot{x}}^{2}\left(k+1\right)-2\hat{\dot{x}}\left(k+1|k\right)\dot{x}\left(k+1\right)\\ y^{2}\left(k+1\right)-2\hat{y}\left(k+1|k\right)y\left(k+1\right)\\ {\dot{y}}^{2}\left(k+1\right)-2\hat{\dot{y}}\left(k+1|k\right)\dot{y}\left(k+1\right) \end{array}\right]+\left[\begin{array}{c} {\hat{x}}^{2}\left(k+1|k\right)\\ {\hat{\dot{x}}}^{2}\left(k+1|k\right)\\ {\hat{y}}^{2}\left(k+1|k\right)\\ {\hat{\dot{y}}}^{2}\left(k+1|k\right) \end{array}\right] \end{array}$$

The first item (in the last line) of the above equation can be written as:

(23) $$\begin{array}{l} \left[\begin{array}{c} x^{2}\left(k+1\right)-2\hat{x}\left(k+1|k\right)x\left(k+1\right)\\ {\dot{x}}^{2}\left(k+1\right)-2\hat{\dot{x}}\left(k+1|k\right)\dot{x}\left(k+1\right)\\ y^{2}\left(k+1\right)-2\hat{y}\left(k+1|k\right)y\left(k+1\right)\\ {\dot{y}}^{2}\left(k+1\right)-2\hat{\dot{y}}\left(k+1|k\right)\dot{y}\left(k+1\right) \end{array}\right]\\ =\left[\begin{array}{c} x^{2}\left(k+1\right)\\ {\dot{x}}^{2}\left(k+1\right)\\ y^{2}\left(k+1\right)\\ {\dot{y}}^{2}\left(k+1\right) \end{array}\right]-2\left[\begin{array}{c} \hat{x}\left(k+1|k\right)x\left(k+1\right)\\ \hat{\dot{x}}\left(k+1|k\right)\dot{x}\left(k+1\right)\\ \hat{y}\left(k+1|k\right)y\left(k+1\right)\\ \hat{\dot{y}}\left(k+1|k\right)\dot{y}\left(k+1\right) \end{array}\right]\\ ={\left(\text{x}\left(k+1\right)\right)}^{2}-2\hat{\text{x}}\left(k+1|k\right)\text{x}\left(k+1\right) \end{array}$$

wherein the multiplication in the last line is also a scalar multiplication. The first item of Equation (23) is a nonlinear function of **x**(*k* + 1). (**x**(*k* + 1))^2^ can further be expanded into a Taylor around **x̂**(*k* + 1∣*k*). However, this procession makes things complicated. According to the knowledge in Kalman filtering, **x**(*k* + 1) is the unbiased estimation of **x**(*k* + 1). Therefore, [*x*^2^(*k* + 1) *ẋ*^2^(*k* + 1) *y*^2^(*k* + 1) *ẏ*^2^(*k* + 1)]*^T^* in Equations (22) and (23) is approximately: ${\left[\hat{x}(k+1|k)\;x(k+1)\;\hat{\dot{x}}(k+1|k)\dot{x}(k+1)\;\hat{y}(k+1|k)\;y(k+1)\;\hat{\dot{y}}(k+1|k)\dot{y}(k+1)\right]}^{T}$.Then,

(24) $$\begin{array}{l} \left[\begin{array}{c} x^{2}\left(k+1\right)\\ {\dot{x}}^{2}\left(k+1\right)\\ y^{2}\left(k+1\right)\\ {\dot{y}}^{2}\left(k+1\right) \end{array}\right]-2\left[\begin{array}{c} \hat{x}\left(k+1|k\right)x\left(k+1\right)\\ \hat{\dot{x}}\left(k+1|k\right)\dot{x}\left(k+1\right)\\ \hat{y}\left(k+1|k\right)y\left(k+1\right)\\ \hat{\dot{y}}\left(k+1|k\right)\dot{y}\left(k+1\right) \end{array}\right]\\ \approx \left[\begin{array}{c} \hat{x}\left(k+1|k\right)x\left(k+1\right)\\ \hat{\dot{x}}\left(k+1|k\right)\dot{x}\left(k+1\right)\\ \hat{y}\left(k+1|k\right)y\left(k+1\right)\\ \hat{\dot{y}}\left(k+1|k\right)\dot{y}\left(k+1\right) \end{array}\right]-2\left[\begin{array}{c} \hat{x}\left(k+1|k\right)x\left(k+1\right)\\ \hat{\dot{x}}\left(k+1|k\right)\dot{x}\left(k+1\right)\\ \hat{y}\left(k+1|k\right)y\left(k+1\right)\\ \hat{\dot{y}}\left(k+1|k\right)\dot{y}\left(k+1\right) \end{array}\right]\\ =-\left[\begin{array}{c} \hat{x}\left(k+1|k\right)x\left(k+1\right)\\ \hat{\dot{x}}\left(k+1|k\right)\dot{x}\left(k+1\right)\\ \hat{y}\left(k+1|k\right)y\left(k+1\right)\\ \hat{\dot{y}}\left(k+1|k\right)\dot{y}\left(k+1\right) \end{array}\right]\\ =-\hat{\text{x}}\left(k+1|k\right)\text{x}\left(k+1\right). \end{array}$$

wherein the multiplication in the last line is a scalar multiplication. Consequently, Equation (21) is shaped into:

(25) $$\begin{array}{l} \text{z}\left(k+1\right)\\ =f\left(\hat{\text{x}}\left(k+1|k\right)\right)+\psi_{1}\left(k\right)\left(\text{x}\left(k+1\right)-\hat{\text{x}}\left(k+1|k\right)\right)\\ +\psi_{2}\left(k\right){\left(\text{x}\left(k+1\right)-\hat{\text{x}}\left(k+1|k\right)\right)}^{2}+\text{w}\left(k+1\right)\\ \approx \left[\psi_{1}\left(k\right)-\psi_{2}\left(k\right)\hat{\text{x}}\left(k+1|k\right)\right]\text{x}\left(k+1\right)\\ +f\left(\hat{\text{x}}\left(k+1|k\right)\right)+\psi_{2}\left(k\right){\hat{\text{x}}}^{2}\left(k+1|k\right)+\text{w}\left(k+1\right). \end{array}$$

Let:

(26) $$\{\begin{array}{l} {\text{C}}_{0}\left(k\right)=\psi_{1}\left(k\right)-\psi_{2}\left(k\right)\hat{\text{x}}\left(k+1|k\right)\\ {\text{w}}_{0}\left(k+1\right)=f\left(\hat{\text{x}}\left(k+1|k\right)\right)+\psi_{2}\left(k\right){\hat{\text{x}}}^{2}\left(k+1|k\right)+\text{w}\left(k+1\right) \end{array}$$

Ignoring the loss in the approximation, Equation (25) can be written as follows:

(27) $$\text{z}\left(k+1\right)={\text{C}}_{0}\left(k\right)\text{x}\left(k+1\right)+{\text{w}}_{0}\left(k+1\right).$$

The above equation is the linearized observation model for “EKF-normal.” **w**_0_(*k* + 1) is the observation noise. The predicting and filtering steps of the “EKF-normal” algorithm are in accordance with Section 3.3.

#### 3.4.3. The Observation Model in a Widely-Used Fast EKF

In Equation (16), ignoring higher powers (than first order) of **x**(*k* + 1) − **x̂**(*k* + 1∣*k*) leads to:

(28) $$\begin{array}{l} \text{z}\left(k+1\right)\\ =f\left(\hat{\text{x}}\left(k+1|k\right)\right)+\psi_{1}\left(k\right)\left(\text{x}\left(k+1\right)-\hat{\text{x}}\left(k+1|k\right)\right)+\text{w}\left(k+1\right)\\ =\psi_{1}\left(k\right)\text{x}\left(k+1\right)+f\left(\hat{\text{x}}\left(k+1|k\right)\right)-\psi_{1}\left(k\right)\hat{\text{x}}\left(k+1|k\right)+\text{w}\left(k+1\right). \end{array}$$

Let:

(29) $$\{\begin{array}{l} {\text{C}}_{1}\left(k\right)=\psi_{1}\left(k\right)\\ {\text{w}}_{1}\left(k+1\right)=f\left(\hat{\text{x}}\left(k+1|k\right)\right)-\psi_{1}\left(k\right)\hat{\text{x}}\left(k+1|k\right)+\text{w}\left(k+1\right) \end{array}.$$

Then, Equation (29) can be written as follows:

(30) $$\text{z}\left(k+1\right)={\text{C}}_{1}\left(k\right)\text{x}\left(k+1\right)+{\text{w}}_{1}\left(k+1\right).$$

This is the linearized observation model for “EKF-fast1.” **w**_1_(*k* + 1) is the observation noise. The predicting and filtering steps of the “EKF-fast1” are in agreement with Section 3.3.

### 3.5. Calculation of the Filtering Gain

One of our previous works has focused on fast algorithms for calculating filtering gain [5]. The fundamental three equations are repeated here for convenience (excerpted, translated and reorganized from our Chinese paper with the permission of all of the authors and the publisher [5]).

In Equation (15), let **M**(*k* + 1) = **C**(*k* + 1)**P**(*k* + 1∣*k*)**C***^T^*(*k* + 1) + **R**(*k* + 1) and **N**(*k* + 1) = **M**(*k* + 1) − **I**. **I** is the identity matrix. Suppose that λ_N,i_ (*i* = 1, 2) are the eigenvalues of **N**(*k* + 1), and λ_N,m_ (1 ≤ m ≤ 2) is the one with larger absolute value. Let λ_M,i_ (*i* = 1, 2) be the eigenvalues of **M**(*k* + 1), and λ_M,m_ (1 ≤ *m* ≤ 2) is the one with larger absolute value.

In order to calculate filtering gain, there are three cases to be considered.

| λ_N_,_m_ | is smaller than one:

In this situation, the power series of **N**(*k* + 1) is convergent. **M**^−1^(*k* + 1) is arrived at by:

(31) $$\begin{array}{ll} {\text{M}}^{-1}\left(k+1\right) & =\frac{1}{\text{M}\left(k+1\right)}\\ & =\frac{1}{\text{I}+\left[\text{M}\left(k+1\right)-\text{I}\right]}\\ & =\frac{1}{\text{I}+\text{N}\left(k+1\right)}\\ & =\text{I}-\text{N}\left(k+1\right)+{\text{N}}^{2}\left(k+1\right)-{\text{N}}^{3}\left(k+1\right)+{\text{N}}^{4}\left(k+1\right)-\cdots . \end{array}$$

λ_N,m_ is larger than one:

In this situation, the power series of **N**(*k* + 1) is divergent. It is obvious that λ_M,m_ > 1 in this case. Let λ_M,m_ < *ρ*_1_ < λ_M,m_ + 1. *ρ*_1_ is a real number. **M**^−1^(*k* + 1) is arrived at by:

(32) $$\begin{array}{ll} {\text{M}}^{-1}\left(k+1\right) & =\frac{1/\rho_{1}}{\text{M}\left(k+1\right)/\rho_{1}}\\ & =\frac{1/\rho_{1}}{\text{I}+[\text{M}\left(k+1\right)-\rho_{1}\text{I}]/\rho_{1}}\\ & =\frac{1}{\rho_{1}}\times \frac{1}{\text{I}+{\text{N}}_{1}\left(k+1\right)}\\ & =\frac{1}{\rho_{1}}\left(\text{I}-{\text{N}}_{1}\left(k+1\right)+{\text{N}}_{1}^{2}\left(k+1\right)-{\text{N}}_{1}^{3}\left(k+1\right)+{\text{N}}_{1}^{4}\left(k+1\right)-\cdots \right) \end{array}$$

wherein **N**_1_(*k* + 1) = [**M**(*k* + 1) − *ρ*_1_**I**] /*ρ*_1_.

|λ_N,m_| is larger than one; λ_N,m_ is negative:

The power series of **N**(*k* + 1) is also divergent in this situation. Let |λ_M,m_| < *ρ*_2_ < |λ_M,m_| + 1. *ρ*_2_ is also a real number. **M**^−1^(*k* + 1) can be arrived at by:

(33) $$\begin{array}{ll} {\text{M}}^{-1}\left(k+1\right) & =\frac{1/\rho_{2}}{\text{M}\left(k+1\right)/\rho_{2}}\\ & =\frac{1/\rho_{2}}{\text{I}+\left[\text{M}\left(k+1\right)+\rho_{2}\text{I}\right]/\rho_{2}}\\ & =\frac{1}{\rho_{2}}\times \frac{1}{\text{I}+{\text{N}}_{2}\left(k+1\right)}\\ & =\frac{1}{\rho_{2}}\left(\text{I}-{\text{N}}_{2}\left(k+1\right)+{\text{N}}_{2}^{2}\left(k+1\right)-{\text{N}}_{2}^{3}\left(k+1\right)+{\text{N}}_{2}^{4}\left(k+1\right)-\cdots \right) \end{array}$$

wherein **N**_2_(*k* + 1) = [**M**(*k* + 1) + *ρ*_2_**I**] /*ρ*_2_.

For all of the above-mentioned three cases, the more items calculated in the power series, a better precision of **M**^−1^ (*k* +1) may be achieved. A better precision of **K**(*k* +1) may thus be realized. However, only the first and the second order of **N**(*k* + 1) (or, **N**_1_(*k* + 1), **N**_2_(*k* + 1), due to the value of λ_N,m_) are considered in EKF-fast1. The first, the second and the third order of **N**(*k* + 1) (or other expressions, due to the value of λ_N,m_) are counted in EKF-normal. The cognition processions of filtering gain in EKF-fast2 will be illustrated in the coming materials.

For all of the EKF algorithms, the result of **M**^−1^(*k* + 1) is further utilized in calculating the filtering gain by Equation (15).

## 4. The Cognition-Based Algorithm to Ease the Computational Load for the Extended Kalman Filter

### 4.1. The Idea of the Algorithm

As can be known from Section 3.4, EKF-fast1 considers less items (than the normal EKF does) in Taylor expansion in linearizing the observation model and calculating the filtering gain. The literature shows that more items may ensure better performance in estimation output [11,18]. However, more items introduce much computational load. The additional item may be unnecessary when the target runs smoothly and only light noise is introduced. Therefore, the key points of our designation for EKF-fast2 are:

- In linearizing the observation model, there are two solutions: one is Equation (27), while the other is Equation (30). If the localizing performance is no worse than the expected performance, Equation (30) is applied. If the localizing performance is worse than the expected performance, Equation (27) is applied.
- In calculating the filtering gain, there are also two approaches. We will illustrate this problem when |λ_N,m_| is smaller than one. The other cases of | λ_N,m_| are similar. If the predicting performance is worse than the expected performance, all of the first order, the second order and the third order of **N**(*k* + 1) in Equation (31) will be taken into account. If the predicting performance is no worse than the expected performance, the first order and the second order of **N**(*k* + 1) in Equation (31) will be calculated.
- The performance is measured with respect to the notion of Euclidean distance. The performance evaluation result is fed back to support the aforementioned decision (on calculation) making.

**Figure 2.**
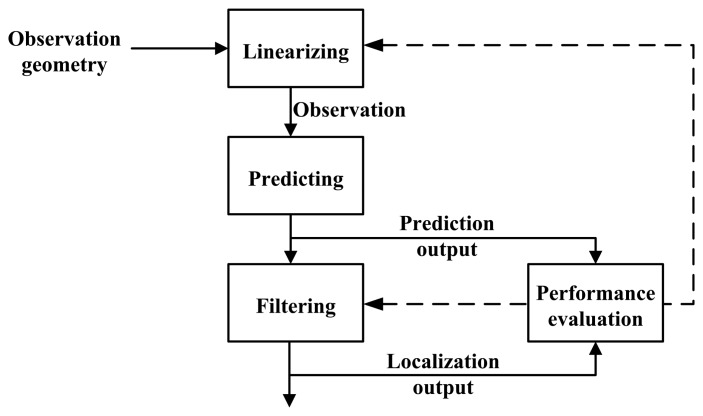
The idea of the cognition-based EKF fast algorithm.

**Figure 3.**
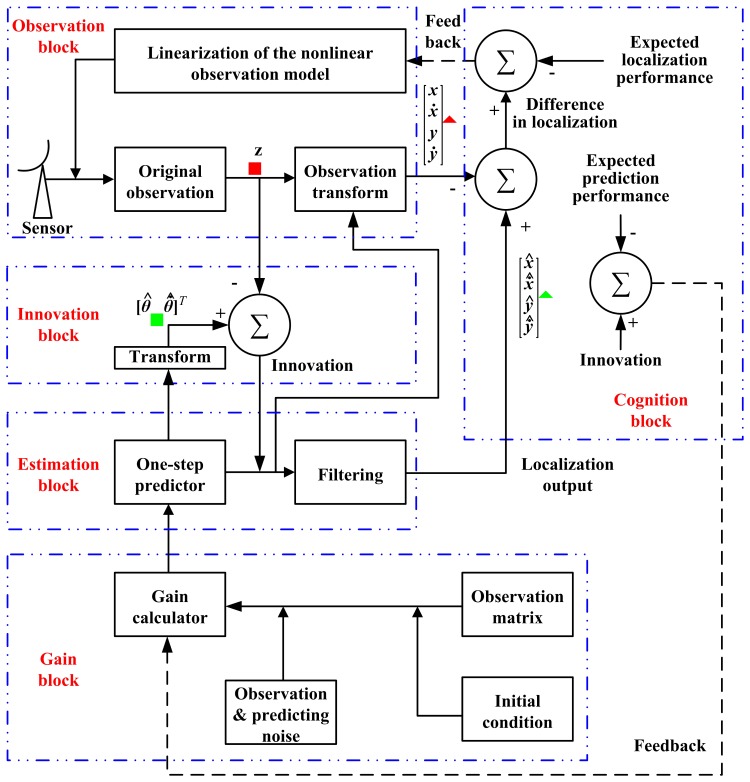
The block diagram for the cognition-based EKF fast algorithm.

The cognition idea is illustrated in Figure 2, while the block diagram is shown in Figure 3.

In these two figures, the forward signal is denoted by a solid line, and the dashed line shows the feedback signal. The signal in the filtering closed-loop is all shown by a solid line. In Figure 3, (1) the **Z**^−1^ module is a delayer that delays the input signal by a data acquisition period; (2) the localizing output from the filtering branch is marked by a green triangle ( 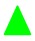), while the localizing output from the observation branch is marked by a red triangle ( 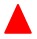); and (3) the predicting output from the filtering branch is marked by a green square ( 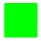), while the data from the observation branch is marked by a red square ( 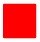).

### 4.2. The Way to Evaluate the Predicting Performance

As mentioned above, the performance is measured with respect to the notion of Euclidean distance.

For the prediction to be employed in performance evaluating, there are two kinds of predicting output: (1) prediction of the target's state; and (2) prediction of the target's azimuth and the rate of change in the azimuth. The difference between these two estimations is only a transform. In the observation block, however, the original observation data are closer to target's true value (azimuth and the rate of change in the azimuth) than the other one (prediction of the target's state in the observation block). Therefore, we evaluate the predicting performance with the prediction of the target's azimuth and the rate of change in the azimuth.

Let $\hat{\theta }(k)={\left[\hat{\theta }(k)\;\hat{\dot{\theta }}(k)\right]}^{T}$ be the predicting output (from the estimation block) of the target's azimuth and the rate of change in the azimuth. The difference between the predicting output and the observation reads:

(34) $${\text{D}}_{\text{Predict}}\left(k\right)=\left[\begin{array}{c} D_{11}\left(k\right)\\ D_{12}\left(k\right) \end{array}\right]=\left[\begin{array}{c} \left|\hat{\theta }\left(k\right)-z_{1}\left(k\right)\right|\\ \left|\hat{\dot{\theta }}\left(k\right)-z_{2}\left(k\right)\right| \end{array}\right]$$

The performance bound for predicting is ***η***_predict_ = [*η*_11_ *η*_12_]*^T^*. The predicting performance is given by:

(35) $$P_{\text{predict}}\left(k\right)=\{\begin{array}{l} \sqrt{{\left(\frac{D_{11}\left(k\right)}{\eta_{11}}\right)}^{2}+{\left(\frac{D_{12}\left(k\right)}{\eta_{12}}\right)}^{2}},\text{if}\;D_{11}\left(k\right)<\eta_{11}\;\text{and}\;D_{12}\left(k\right)<\eta_{12}\\ 2,\text{otherwise} \end{array}.$$

*P*_predict_(*k*) will be further used to make a decision on choosing the filtering gain calculating models.

### 4.3. The Way to Evaluate the Localizing Performance

#### 4.3.1. The “Localizing” Techniques in the Observation Block

As was presented previously, only the azimuth and the rate of change in the azimuth of the target are observed. To know the difference between the localizing output from the estimation block and the observation block, the target's location is resolved from the observation data. Here, we call the subtraction result “difference” instead of “error.” This is because there is an error in both of the estimations (localization based on observation and localization with filtering) when performing the localizing work. However, most of the existing literature has not mentioned this point. The conventional technique is taking (1) the localizing output based on observation as (2) the true state of the target. Readers should notice the difference in the physical properties of these two variables. In this paper, the above-mentioned subtraction result is called “error” only in the experiments, which may be familiar for the readers.

Determination of the position of the target:

As can be obtained from Equation (6),

(36) $$\frac{y\left(k+1\right)}{x\left(k+1\right)}=\tan\left[\theta \left(k+1\right)\right].$$

Combining Equations (36) and (7) gives:

(37) $$\{\begin{array}{l} \frac{y\left(k+1\right)}{x\left(k+1\right)}=\tan\left[\theta \left(k+1\right)\right]\\ \frac{\dot{x}\left(k+1\right)y\left(k+1\right)-\dot{y}\left(k+1\right)x\left(k+1\right)}{x^{2}\left(k+1\right)+y^{2}\left(k+1\right)}=\dot{\theta }\left(k+1\right) \end{array}$$

There are four variables to be resolved in Equation (37). To obtain the position for the target, some approximations are introduced. In the data collecting course, *ẋ*(*k* + 1) is approximately:

(38) $$\dot{x}\left(k+1\right)\approx \frac{x\left(k+1\right)-x\left(k\right)}{\Delta t}$$

*ẏ*(*k* + 1) is approximately:

(39) $$\dot{y}\left(k+1\right)\approx \frac{y\left(k+1\right)-y\left(k\right)}{\Delta t}$$

The approximations are excellent as long as the data acquisition period is short and the target's maximum incremental change in acceleration between any two observations is limited. Of course, there is some variation in these approximations due to the specific scenario, but this approximation serves as a good estimation. Moreover, $\hat{\dot{x}}(k+1|k)$ is the unbiased estimation of *ẋ*(*k* + 1), and $\hat{\dot{y}}(k+1|k)$ is the unbiased estimation of *ẏ*(*k* + 1), respectively. Consequently,

(40) $$\{\begin{array}{l} \frac{y\left(k+1\right)}{x\left(k+1\right)}=\tan\left[\theta \left(k+1\right)\right]\\ \frac{\hat{\dot{x}}\left(k+1|k\right)y\left(k+1\right)-\hat{\dot{y}}\left(k+1|k\right)x\left(k+1\right)}{x^{2}\left(k+1\right)+y^{2}\left(k+1\right)}\approx \dot{\theta }\left(k+1\right) \end{array}$$

Ignoring the loss in the approximation results in:

(41) $$\{\begin{array}{l} \frac{y\left(k+1\right)}{x\left(k+1\right)}=\tan\left[\theta \left(k+1\right)\right]\\ \frac{\hat{\dot{x}}\left(k+1|k\right)y\left(k+1\right)-\hat{\dot{y}}\left(k+1|k\right)\left(k+1\right)}{x^{2}\left(k+1\right)+y^{2}\left(k+1\right)}=\dot{\theta }\left(k+1\right) \end{array}$$

Then, *y*(*k* + 1) and *x*(*k* + 1) are the solutions to Equation (41),

(42) $$\{\begin{array}{l} x\left(k+1\right)=\frac{\left\{\hat{\dot{x}}\left(k+1|k\right)-\hat{\dot{y}}\left(k+1|k\right)\tan\left[\theta \left(k+1\right)\right]\right\}\tan\left[\theta \left(k+1\right)\right]}{\dot{\theta }\left(k+1\right)\left\{\tan^{2}\left[\theta \left(k+1\right)\right]+1\right\}}\\ y\left(k+1\right)=\frac{\hat{\dot{x}}\left(k+1|k\right)-\hat{\dot{y}}\left(k+1|k\right)\tan\left[\theta \left(k+1\right)\right]}{\dot{\theta }\left(k+1\right)\left\{\tan^{2}\left[\theta \left(k+1\right)\right]+1\right\}} \end{array}$$

Determination of velocity for the target:

Like the processions mentioned previously, some approximations are introduced in this procedure. In solving *ẋ*(*k* + 1), *ẏ*(*k* + 1) is approximately $\hat{\dot{y}}(k+1|k)$. Similarly, in finding *ẏ*(*k* + 1), *ẋ*(*k* + 1) is approximately $\hat{\dot{x}}(k+1|k)$. Thus,

(43) $$\{\begin{array}{l} \frac{\dot{x}\left(k+1\right)y\left(k+1\right)-\hat{\dot{y}}\left(k+1|k\right)x\left(k+1\right)}{x^{2}\left(k+1\right)+y^{2}\left(k+1\right)}\approx \dot{\theta }\left(k+1\right)\\ \frac{\hat{\dot{x}}\left(k+1|k\right)y\left(k+1\right)-\dot{y}\left(k+1\right)x\left(k+1\right)}{x^{2}\left(k+1\right)+y^{2}\left(k+1\right)}\approx \dot{\theta }\left(k+1\right) \end{array}$$

Ignoring the minor error in the approximation gives:

(44) $$\{\begin{array}{l} \frac{\dot{x}\left(k+1\right)y\left(k+1\right)-\hat{\dot{y}}\left(k+1|k\right)x\left(k+1\right)}{x^{2}\left(k+1\right)+y^{2}\left(k+1\right)}=\dot{\theta }\left(k+1\right)\\ \frac{\hat{\dot{x}}\left(k+1|k\right)y\left(k+1\right)-\dot{y}\left(k+1\right)x\left(k+1\right)}{x^{2}\left(k+1\right)+y^{2}\left(k+1\right)}=\dot{\theta }\left(k+1\right) \end{array}$$

Solving Equation (44) yields:

(45) $$\{\begin{array}{l} \dot{x}(k+1)=\frac{\dot{\theta }(k+1)[x^{2}(k+1)+y^{2}(k+1)]+\hat{\dot{y}}(k+1|k)x(k+1)}{y(k+1)}\\ \dot{y}(k+1)=\frac{\hat{\dot{x}}(k+1|k)y(k+1)-\dot{\theta }(k+1)\left[x^{2}(k+1)+y^{2}(k+1)\right]}{x(k+1)} \end{array}$$

Using the results in Equation (42) leads to:

(46) $$\{\begin{array}{l} \dot{x}(k+1)=\frac{\varsigma {\dot{\theta }}^{2}(k+1)\{\tan^{2}[\theta (k+1)]+1\}}{\hat{\dot{x}}(k+1|k)-\hat{\dot{y}}(k+1|k)\tan[\theta (k+1)]}+\xi \hat{\dot{y}}(k+1|k)\\ \dot{y}(k+1)=\frac{1}{\xi }\hat{\dot{x}}(k+1|k)-\frac{\varsigma {\dot{\theta }}^{2}(k+1)\{\tan^{2}[\theta (k+1)]+1\}}{\left\{\hat{\dot{x}}(k+1|k)-\hat{\dot{y}}(k+1|k)\tan[\theta (k+1)]\}\tan[\theta (k+1)\right]} \end{array}$$

where:

(47) $$\begin{array}{ll} \varsigma & =x^{2}(k+1)+y^{2}(k+1)\\ & =\frac{{\left\{\hat{\dot{x}}(k+1|k)-\hat{\dot{y}}(k+1|k)\tan[\theta (k+1)]\right\}}^{2}\tan^{2}[\theta (k+1)]+{\left\{\hat{\dot{x}}(k+1|k)-\hat{\dot{y}}(k+1|k)\tan[\theta (k+1)]\right\}}^{2}}{{\left[\dot{\theta }(k+1)\right]}^{2}{\left\{\tan^{2}[\theta (k+1)]+1\right\}}^{2}} \end{array}$$

and:

(48) $$\begin{array}{ll} \xi & =\frac{x(k+1)}{y(k+1)}\\ & =\frac{\left\{\hat{\dot{x}}(k+1|k)-\hat{\dot{y}}(k+1|k)\tan[\theta (k+1)]\}\tan[\theta (k+1)\right]}{\hat{\dot{x}}(k+1|k)-\hat{\dot{y}}(k+1|k)\tan[\theta (k+1)]} \end{array}.$$

With Equations (42) and (46), the target's position and velocity are estimated according to the observation data. In this course, there is information support $\left(\hat{\dot{x}}(k+1|k),\;\hat{\dot{y}}(k+1|k)\right)$ from the estimation block, as is shown in Figure 3. It should be noted that there is observation noise in the observing course. Therefore, when calculating **x**(*k*) with (42) and Equation (46)*z*_1_(*k*) will be substituted for *θ*(*k*), while *θ̇*(*k*) will be replaced by *z*_2_(*k*).

#### 4.3.2. The Way to Evaluate the Performance in Localizing

The difference between the filtering output and the localizing result via the observation reads:

(49) $$D_{\text{localize}}\left(k\right)=\left[\begin{array}{c} D_{21}\left(k\right)\\ D_{22}\left(k\right)\\ D_{23}\left(k\right)\\ D_{24}\left(k\right) \end{array}\right]=\left[\begin{array}{c} \left|\hat{x}\left(k|k\right)-x\left(k\right)\right|\\ \left|\hat{\dot{x}}\left(k|k\right)-\dot{x}\left(k\right)\right|\\ \left|\hat{y}\left(k|k\right)-y\left(k\right)\right|\\ \left|\hat{\dot{y}}\left(k|k\right)-\dot{y}\left(k\right)\right| \end{array}\right]$$

The performance bound for localizing is ***η***_localize_ = [*η*_21_ *η*_22_ *η*_23_ *η*_24_]*^T^*. The localizing performance is given by:

(50) $$P_{\text{localize}}\left(k\right)=\{\begin{array}{l} \sqrt{\sum_{\text{i}=1}^{4}{\left(\frac{D_{2i}\left(k\right)}{\eta_{2i}}\right)}^{2}},\;\text{if}\;D_{2i}\left(k\right)<\eta_{2i},i=1,2,3,4\\ 2,\text{otherwise} \end{array}$$

*P*_localize_(*k*) will be further used to configure the method in linearizing the observation model.

### 4.4. The Cognition Processing

On the basis of the aforementioned work, the cognition processing is easy implement.

The way to ease the computational load in linearizing the observation equation:

In linearizing the observation model, if *P*_localize_(*k*) is smaller than two, then the observation Equation (3) is linearized according to Equation (30); if *P*_localize_(*k*) is equal to two, then the observation Equation (3) is linearized with respect to Equation (27).

The approach to ease the computational load in determining the filtering gain:

According to the value of λ_N,m_ (1 ≤ *m* ≤ 2), one of Equations (31)–(33) is chosen to compute the filtering gain in EKF-fast2.

In calculating the filtering gain, if *P*_predict_(*k*) is smaller than two, then only the first and the second order of **N**(*k* + 1) (or **N**_1_(*k* + 1), **N**_2_(*k* + 1), due to the value of λ_N,m_) are considered; if *P*_predict_(*k*) equals two, the first, the second and the third order of **N**(*k* + 1) (or other expressions, due to the value of λ_N,m_) are all calculated.

**Figure 4.**
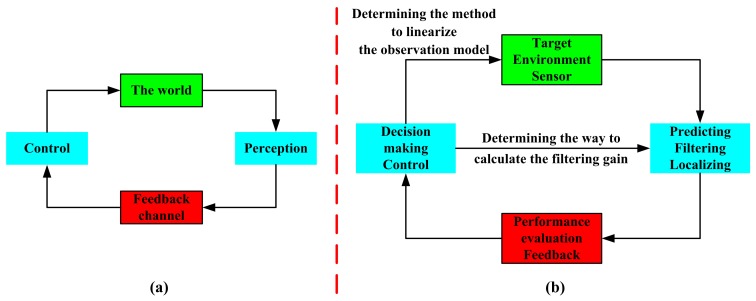
(**a**) The cognition notion in a cognitive radar; (**b**) the cognition mechanism in this work.

A summary of the cognition mechanism:

As can be known from all of the above-mentioned materials, the cognition processing focuses on three steps: (1) perception; with the mentioned extended Kalman filter, the azimuth and the rate of change in the azimuth of the target are predicted, and the position of the target is estimated (Section 3.3 and Section 4.3.1); (2) feedback; the prediction performance and the localization performance are evaluated (Section 4.2 and Section 4.3.2); (3) control; according to the forwarded performance evaluation result, the system makes decisions on the method to linearize the observation model and the way to calculate the filtering gain (Section 4.4). These are presented in Figure 4.

As shown in Figure 4a, for an active radar, the cognitive cycle is: perceiving the world (target and its environment) with a sensor, signal processing with the use of prior knowledge (as well as learning through interactions with the environment), feeding back of the performance, and adapting both its receiver and transmitter in response to statistical variations in the environment in real time [25,26]. Figure 4b shows the realization (in this work) of the corresponding block in Figure 4a, as is described in the former paragraph. It should be noted that this cognitive processing method is effective not only for an active radar, but also useful for a passive radar.

### 4.5. Summary of the Proposed Algorithm

The processing techniques with the proposed algorithm are summarized as follows:

(1) In the initialization phase, the observation model linearizing method and the approach in calculating the filtering gain are in accordance with those in the EKF-normal.
(2) In the working phase, the localizing performance is evaluated (in each step). The result is fed back to decide which observation model is to be utilized at the next step. The predicting performance is measured (in each step), and the result is fed back to choose the calculating model for the filtering gain at the next step.
(3) The other processions are in agreement with those in Section 3.3.

## 5. Experiments

To know the performance of the proposed algorithm, experiments are performed. There are two scenarios taken into account: tracking a model aircraft (Scenario 1) and tracking a car on the ground (Scenario 2). In these, all of the above-mentioned algorithms are checked.

The employed ground-based radar is a passive radar (more accurately, it can be described as an electronic surveillance measurement system). It can offer the observation of the target's azimuth and the rate of change in the azimuth. The radar is installed on a hill, so that there is no barrier between the radar and the target. The target is considered as a point-object.

### 5.1. Tracking a Model Aircraft

#### 5.1.1. Experiment Setup

The target:

In Scenario 1, the target is a model aircraft with an FM (frequency modulation) transmitter. The radio frequency is 3.5 GHz. The target is voyaging in a three-dimensional plane. The true track of the target is unknown. Only the observations are recorded.

**Figure 5.**
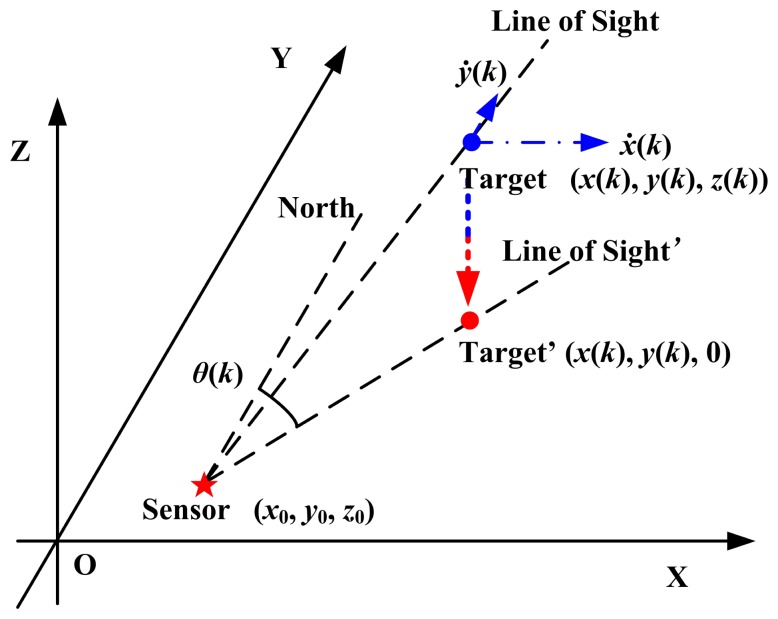
The radar-target geometry in this experiment.

The observation geometry:

Since the target is moving in a three-dimensional plane, the observation geometry is different from the settings in Section 3.2. The target is projected onto the XoY plane. The azimuth is the angle between the line of sight and the north. The observation geometry is presented in Figure 5. With no loss of generality, the radar's coordinates are set to (0, 0, 0) in this work.

The observation noise:

The observation noise (introduced by the sensor and the propagation effects) for these three algorithms is identical. To ensure the whitening effect of the observation noise in the experiment, the noise is generated by the summation of three independent and identically distributed (i.i.d.) Gaussian noises, whose mean is zero and whose variance is each one. Therefore,

(51) $$\begin{array}{ll} \text{w}\left(k\right) & ={\left[w_{\theta }\left(k\right)\;w_{\dot{\theta }}\left(k\right)\right]}^{T}\\ & ={\left[\sum_{n=1}^{3}w_{n,1}\left(k\right)\;\sum_{n=1}^{3}w_{n,2}\left(k\right)\right]}^{T} \end{array}$$

where:

(52) $$w_{n,j}\left(k\right)∼N\left(0,1\right),\;n=1,2,3;\;j=1,2.$$

For different algorithms, the actual observation noise is calculated according to the corresponding observation model.

The expected performance:

The expected values of performance metrics for all of the algorithms are:

- Azimuth, 0.2°; the rate of change in the azimuth, 0.007°/s.
- X position, 3 m; X velocity, 5 m/s.
- Y position, 3 m; Y velocity, 5 m/s.

The data rate:

The data acquisition period is 0.1 s for the radar. The data collection work continued 500 s in total.

#### 5.1.2. Simulation Results

**Figure 6.**
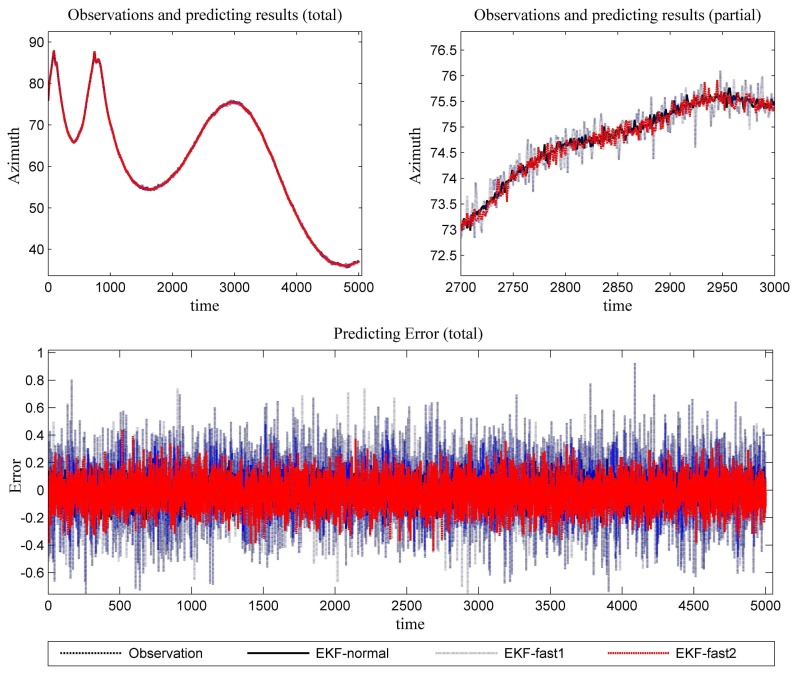
The predicting results (the azimuth and the error in azimuth prediction).

**Figure 7.**
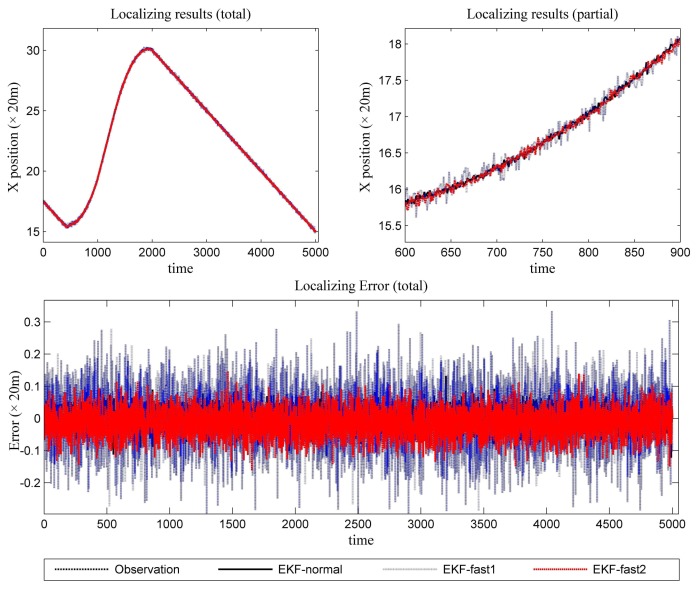
The localizing results (X position and the error in the X position estimation).

**Figure 8.**
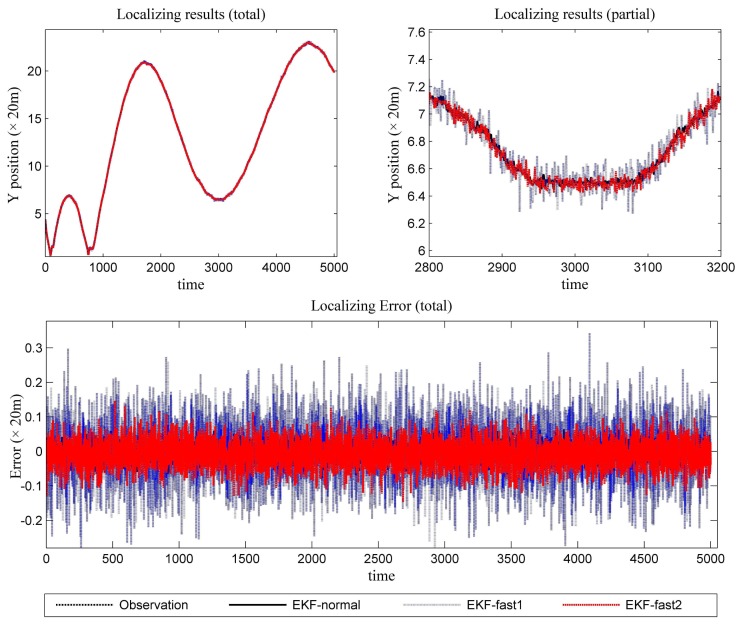
The localizing results (Y position and the error in the Y position estimation).

Figure 6 is the estimation results for the azimuth. The filtering results in X position are presented in Figure 7, while the filtering results in the Y position are shown in Figure 8. In these figures: (1) the “error” means “root-mean-square error (RMSE)”; (2) the “observations” are the transformed observations, called “localization based on observation” in the aforementioned texts; and (3) the “localizing results” are the localizing outputs with filtering. These are also true in the next experiment.

To know the difference in computational requirements, the time consumption is recorded for each mentioned algorithm in the simulation. The data are presented in Table 1. These results were achieved on a Windows PC with an Intel(R) Celeron(R) CPU 430 with a 1.80-GHz processor. The elapsed time is measured in seconds. Only the filtering time of the algorithms is considered. The duration on sensor orientation, noise and clutter outcome, observation accessing and results output have not been included.

#### 5.1.3. Results Analysis

With the data in Figures 6, 7 and 8 and Table 1, we analyze the simulation results with respect to the scenario.

(1) Scenario 1 introduces light noise. As a result, all of the mentioned EKF algorithms work well. The error is acceptable. To achieve a more in-depth understanding of the algorithms, we perform some analysis for the error series. First, the variance of the error series is calculated. Then, the mean of the absolute value for the error series is resolved. The data are presented in Table 2. It is clear that the novel algorithm outperforms the conventional algorithm in estimation work. In light of performance metrics besides the value of RMSE, especially in the aspect of variance, EKF-fast2 is much better than EKF-fast1.
(2) Since this experiment involves light noise, EKF-normal and EKF-fast2 can achieve the expected performance throughout almost the entire test. The precision of EKF-fast1 is not ideal, especially at the maneuvering occasions.
(3) According to Table 1, the ratios between the elapsed time are:
  EKF-fast1:EKF-normal = 54.16%; and EKF-fast2:EKF-normal = 59.70%.

### 5.2. Tracking a Car on the Ground

#### 5.2.1. Experiment Setup

The target:

A car, namely, the target of interest, is running on a plane. There is a radio transmitter on the car. The radio frequency is 10 GHz. The target is a non-cooperative target. Hence, the programmed track of the target is unknown. Only the observations are recorded.

The observation geometry:

The observation geometry is in accordance with the settings in Section 3.2.

The observation noise:

In Scenario 2, the observation noise is subject to the settings in Section 5.1.1. However, the noise is heavier than the noise in Scenario 1. Therefore, in Equation (52)*w_n,j_*(*k*) ∼ *N*(0, 6), *n* = 1, 2, 3; *j* = 1, 2.

The expected performance:

The expected values of performance metrics for all of the algorithms are:

- Azimuth, 0.5°; the rate of change in the azimuth, 0.01°/s.
- X position, 12 m; X velocity, 10 m/s.
- Y position, 12 m; Y velocity, 10 m/s.

The data rate:

The data acquisition period is 1 s for the radar. The data collection work continued about 3500 s in total.

#### 5.2.2. Simulation Results

**Figure 9.**
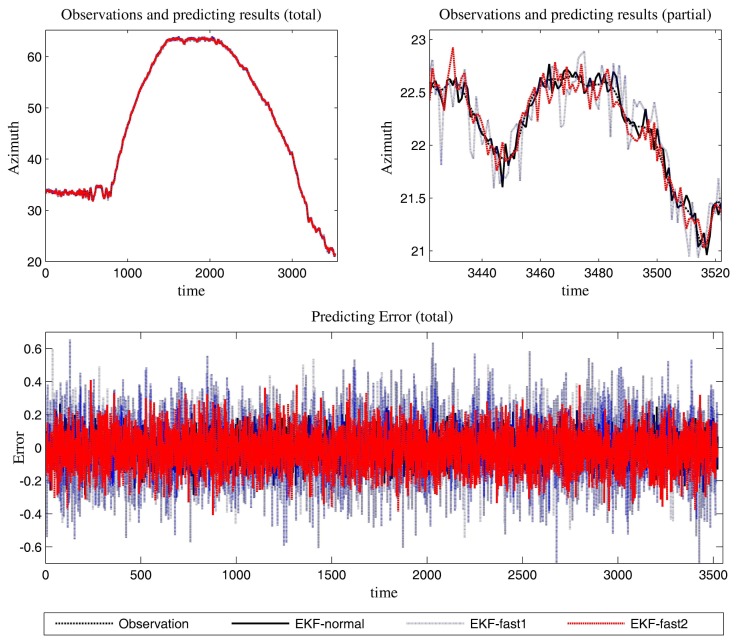
The predicted results (azimuth and the error in azimuth prediction).

The estimation results of the azimuth are presented in Figure 9.

**Figure 10.**
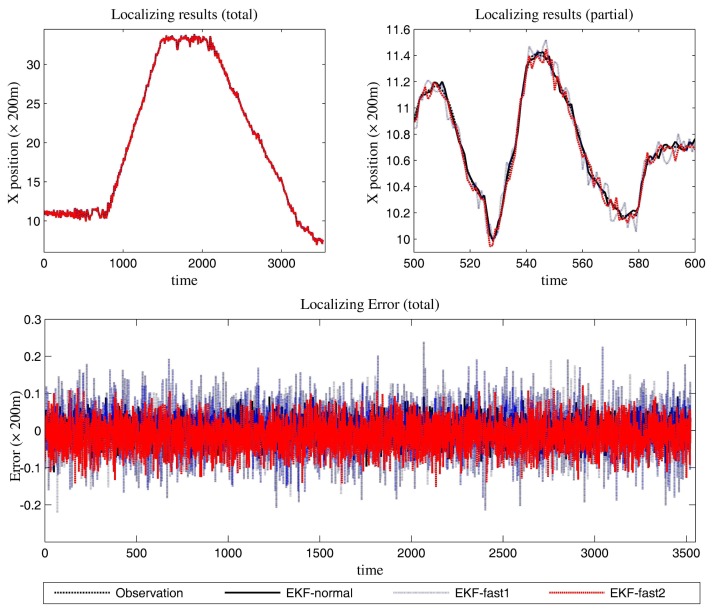
The localizing results (X position and the error in the X position estimation).

**Figure 11.**
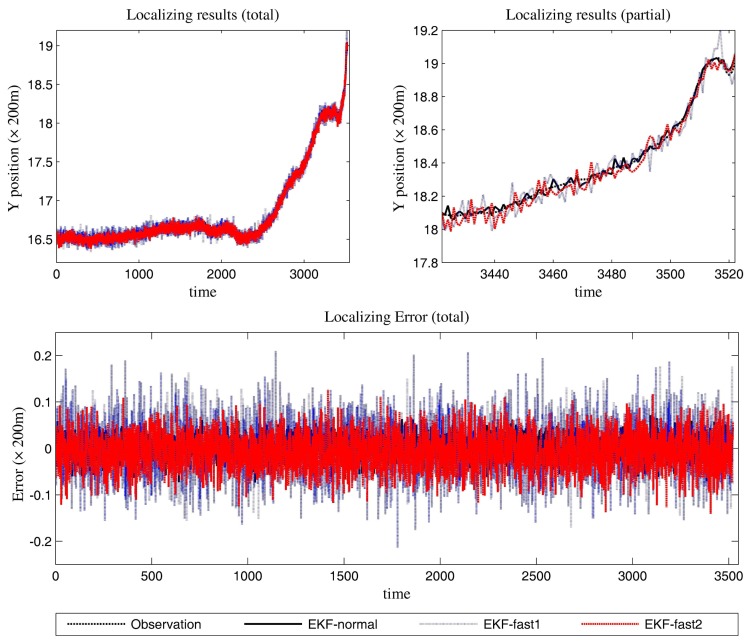
The localizing results (Y position and the error in the Y position estimation).

In Figure 10, the filtering results for the X position are presented. In Figure 11, the filtering results for the Y position are shown.

The time consumption is presented in Table 3. The computer and the method for measuring elapsed time are identical with the former experiment.

#### 5.2.3. Results Analysis

We can gain knowledge from the provided data in Figures 9, 10 and 11 and Table 3:

(1) This scenario brings difficulties for the tracking algorithms. However, the mentioned EKF algorithms can all be applied in target tracking. Compared to the performance in Scenario 1, the noise and the target's maneuvering behavior complicate the algorithms. This results in the tracking performance going down for all of the approaches.
  The in-depth performance data are shown in Table 4. Furthermore, the novel algorithm outperforms the conventional algorithm in estimation work.
(2) The normal EKF (EKF-normal) has the best performance in precision. The existing fast EKF (EKF-fast1) has the least computational requirements. The proposed method (EKF-fast2) has similar performance to EKF-normal. At the same time, the computational load of EKF-fast2 is at a consistent level, as the EKF-fast1 maintains. The reasons for the latter point are plentiful. On the one hand, the cognition processing and the occasionally introduced higher-order Taylor expansion in the linearization bring more computational load to EKF-fast2 than EKF-fast1. On the other hand, EKF-fast2 improves the computation work (compared to EKF-fast1) in resolving the filtering gain.
(3) According to Table 3, the ratios between the elapsed time are:
  EKF-fast1:EKF-normal = 57.17%; and EKF-fast2:EKF-normal = 60.10%.

Since there are only 2 × 4 entries in **C**(*k*) (**C**_0_(*k*), **C**_1_(*k*) and **C**_2_(*k*)) and 2 × 2 entries in **M**(*k*), the improvement in lowering the computational requirements is limited. The improvement in complicated applications may be more substantial.

### 5.3. Comparison between the Existing Technologies and the Proposed Methodology Based on the Simulation Results

Based on the simulation results presented above, a comparison between the existing algorithms and the proposed algorithm is performed in Table 5. The meanings of some symbols are listed below.

- **L1**:Is the algorithm flexible for a complicated scenario?
- **L2**: The precision of the method.
- **L3**: The computational load of the method.
- **L4**: The generalization of the method.
- **L5**: Is the method easy to configure?

According to the information in Table 5, EKF-fast2 is the best choice (among these three methods) for tracking a maneuvering target in a complicated scenario. The observation model is a nonlinear observation model, either an active radar or a passive radar.

## 6. Conclusions

In this paper, a new fast EKF algorithm is proposed. At the same time, the novel method is verified through simulations. The advantages of this technology are that it is flexible and the cognition capability exists. Compared to the EKF-normal algorithm, it works with better efficiency in an identical scenario. The proposed approach is very simple and yet effective. This cognition processing is especially useful for a passive radar. However, some facts must be admitted here. If the target runs smoothly and only light noise is introduced, then the use of this designation is somewhat exaggerated.

In the future, implementing this designation in radar systems will be considered.

## Figures and Tables

**Table 1.** The elapsed time (in seconds) of the mentioned target tracking algorithms.

	**EKF-normal**	**EKF-fast1**	**EKF-fast2**
**Time**	18.1952	10.3964	10.8629

**Table 2.** Comparison of the performance for the mentioned target tracking algorithms.

	**EKF-normal**	**EKF-fast1**	**EKF-fast2**
**Variance of the error in the azimuth estimation**	0.0059	0.0548	0.0133
**Variance of the error in the X position estimation**	0.3528	3.3555	0.6730
**Variance of the error in the Y position estimation**	0.1855	3.0105	0.6235
**Mean of the absolute value for the error series (azimuth estimation)**	0.0731	0.1870	0.0961
**Mean of the absolute value for the error series (X position estimation)**	0.4714	1.4659	0.6867
**Mean of the absolute value for the error series (Y position estimation)**	0.3506	1.3860	0.6407

**Table 3.** The elapsed time (in seconds) of the mentioned target tracking algorithms.

	**EKF-normal**	**EKF-fast1**	**EKF-fast2**
**Time**	12.7461	7.2870	7.6607

**Table 4.** Comparison of the performance of the mentioned target tracking algorithms.

	**EKF-normal**	**EKF-fast1**	**EKF-fast2**
**Variance of the error in the azimuth estimation**	0.0062	0.0387	0.0095
**Variance of the error in the X position estimation**	35.5338	160.4177	68.2008
**Variance of the error in the Y position estimation**	19.1003	137.1015	59.7653
**Mean of the absolute value for the error series (azimuth estimation)**	0.0624	0.1547	0.0970
**Mean of the absolute value for the error series (X position estimation)**	4.7552	10.3763	6.9058
**Mean of the absolute value for the error series (Y position estimation)**	4.4646	9.2098	6.4645

**Table 5.** The comparison between the existing technologies and the proposed methodology.

**Aspect**	**EKF-normal**	**EKF-fast1**	**EKF-fast2**
**L1**	×	×	✓
**L2**	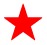	■	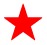
**L3**	■	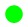	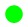
**L4**	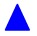	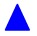	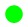
**L5**	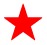	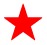	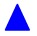

Legend: The following symbols refer to: 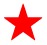, high achievement; 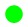, satisfactory; 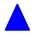, improvement needed; ■, unsatisfactory; ✓, yes; ×, no.
